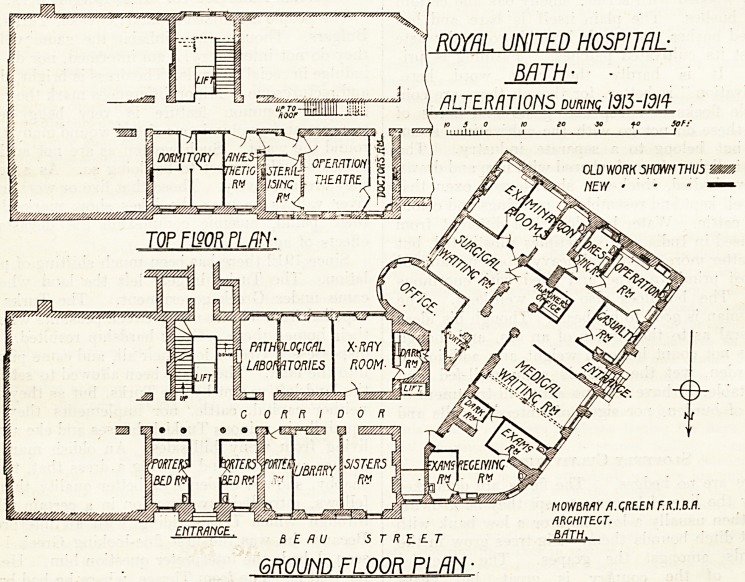# Royal United Hospital, Bath

**Published:** 1916-09-16

**Authors:** 


### HOSPITAL ARCHITECTURE AND CONSTRUCTION.

### Royal United Hospital, Bath.

### ALTERATIONS AND ADDITIONS.

THE alterations carried out during 1913-14 at this hospital affect the top floor and the ground floor.

On the top floor the room adjoining the theatre on the east side was formerly used both for the administration of anæsthetics and for sterilising. A part of this room has been separated by a partition to form a lobby, and the remainder is now used only for sterilising. Part of the adjoining dormitory has been separated by a partition, and now serves as anæsthetic-room with lobby giving access to the theatre.

A portion of the theatre has been cut off by a glass screen, and provides dressing space for surgeons. It is also used by doctors to see an operation without being actually in the theatre.

The heating and ventilation of the department have been entirely remodelled.

The ground-floor alterations consist in an entire remodelling of the out-patient department, which was formerly divided into two parts, one part being in the nursing home on the opposite side of the road.

The entrance, which apparently serves also as an exit, opens into a small inner hall, where, facing the patients as they come in, is the almoner's office. To the left is the medical department, to the right the casualty-room, and beyond the almoner's office is the surgical department.

The medical department comprises a waiting-room, receiving-room, dark-room, and two examining-rooms. The receiving-room answers presumably to what is usually called the consulting-room.

The surgical suite comprises a waiting-room, consulting-room with an examination-room on each side, a surgical dressing-room, the operation-room, and the casualty-room.

An *x*-ray room, with a semi-circular dark-room attached, adjoins the office, which seems somewhat out of place in the middle of the out-patient work; next to the *x*-ray room are two pathological laboratories.

The dispensary has been transferred to the nurses' home on the opposite side of the road.

The position of the dispensary and the fact of there being a common entrance and exit for the patients are the only blots to be noted in an otherwise excellent bit of remodelling.

In the basement a new bath for mineral-water bathing has been installed; a new boiler-house has been erected; and the heating and hot-water services remodelled and improved. In connection with these improvements is included the provision of wash-basins for the medical staff in all the wards.

The whole of the works were carried out under the direction of Mr. Mowbray A. Green, F.R.I.B.A., of Bath.

## Figures and Tables

**Figure f1:**